# Real-world effectiveness of nivolumab plus ipilimumab and second-line therapy in Japanese untreated patients with metastatic renal cell carcinoma: 2-year analysis from a multicenter retrospective clinical study (J-cardinal study)

**DOI:** 10.1093/jjco/hyac124

**Published:** 2022-08-03

**Authors:** Takahiro Kojima, Renpei Kato, Tomokazu Sazuka, Hayato Yamamoto, Shohei Fukuda, Kazutoshi Yamana, Noboru Nakaigawa, Yusuke Sugino, Shuzo Hamamoto, Hiroaki Ito, Hiroshi Murakami, Wataru Obara

**Affiliations:** Department of Urology, Faculty of Medicine, University of Tsukuba, Ibaraki, Japan; Department of Urology, Aichi Cancer Center Hospital, Aichi, Japan; Department of Urology, Iwate Medical University, Iwate, Japan; Department of Urology, Chiba University Graduate School of Medicine, Chiba, Japan; Department of Urology, Hirosaki University Graduate School of Medicine, Aomori, Japan; Department of Urology, Tokyo Medical and Dental University, Tokyo, Japan; Department of Urology, Molecular Oncology, Graduate School of Medicine and Dental Sciences, Niigata University, Niigata, Japan; Department of Urology, Yokohama City University Graduate School of Medicine, Kanagawa, Japan; Department of Nephro-Urologic Surgery and Andrology, Mie University Graduate School of Medicine, Mie, Japan; Department of Nephro-urology, Nagoya City University Graduate School of Medical Sciences, Aichi, Japan; Oncology Medical, Bristol-Myers Squibb K.K., Tokyo, Japan; Oncology Medical Affairs, Ono Pharmaceutical Co., Ltd., Tokyo, Japan; Department of Urology, Iwate Medical University, Iwate, Japan

**Keywords:** nivolumab, ipilimumab, renal cell carcinoma, real-world, Japan

## Abstract

**Background:**

Nivolumab plus ipilimumab combination therapy is one of the standard therapies for untreated renal cell carcinoma patients with an International Metastatic Renal Cell Carcinoma Database Consortium intermediate/poor risk. We have previously reported the 1-year analysis results of the effectiveness and safety of nivolumab plus ipilimumab combination therapy in the real-world setting in Japan. Here, we report the effectiveness of nivolumab plus ipilimumab combination therapy and of second-line therapy, using 2-year analysis.

**Methods:**

This retrospective observational study enrolled Japanese patients with previously untreated metastatic renal cell carcinoma who initiated nivolumab plus ipilimumab combination therapy between August 2018 and January 2019. Data were collected from patients’ medical records at baseline and at 3 months, 1 year and 2 years after the last enrollment.

**Results:**

Of the 45 patients enrolled, 10 patients (22.2%) each had non-clear cell renal cell carcinoma and Eastern Cooperative Oncology Group performance status ≥2 at baseline. Median follow-up period was 24.0 months; objective response rate was 41.5%, with 6 patients achieving complete response; median progression-free survival was 17.8 months and 24-month progression-free survival and overall survival rates were 41.6 and 59.1%, respectively. Second-line therapy achieved an objective response rate of 20%; median progression-free survival was 9.8 months. Median progression-free survival 2 was 26.4 months.

**Conclusions:**

The effectiveness of nivolumab plus ipilimumab combination therapy at 2-year analysis in the real-world setting in Japan was comparable to that reported in CheckMate 214. The current analysis also demonstrated the effectiveness of second-line therapy after nivolumab plus ipilimumab combination therapy.

## Teaser

The effectiveness of nivolumab plus ipilimumab for untreated renal cell carcinoma patients in the real-world setting in Japan at 2-year analysis was comparable to that of CheckMate 214.

## Introduction

Treatment of renal cell carcinoma (RCC) has progressed through the cytokine era and target therapy (TT) era and is currently in the immuno-oncology (IO) era. IO therapies that were significantly more effective than the comparator in phase 3 studies included, as first-line therapy, nivolumab (NIVO) + ipilimumab (IPI) (CheckMate 214 study) (1), pembrolizumab+axitinib (KEYNOTE 426 study) (2), avelumab+axitinib (JAVELIN Renal 101 study) (3), NIVO+cabozantinib (CheckMate 9ER study) (4) and pembrolizumab+lenvatinib (CLEAR study) (5). NIVO monotherapy (CheckMate 025 study) (6) was used as second- or later-line therapy.

NIVO+IPI combination therapy (NIVO+IPI) is one of the standard therapies for International Metastatic Renal Cell Carcinoma Database Consortium (IMDC) intermediate/poor risk RCC patients. NIVO+IPI was approved in Japan in August 2018, for IMDC intermediate/poor risk unresectable or metastatic RCC (mRCC) based on the results of CheckMate 214 (2). The 5-year follow-up data of CheckMate 214 have been published, with durable progression-free survival (PFS; 5-year PFS rate, 31%) (7). However, CheckMate 214 included only 38 Japanese patients (intermediate/poor risk, 31 patients) and excluded patients with non-clear cell RCC (nccRCC) and those with poor performance status (PS). In addition, CheckMate 214 did not evaluate the efficacy of second- or later-line therapy following NIVO+IPI. Effectiveness data of sequential therapy such as that for time to second progression (PFS2) may be useful when considering the treatment strategy for RCC from first-line therapy (8,9). Although several studies have reported the effectiveness of TTs after IO therapies (10–16), the optimal second-line therapy after each first-line therapy has not been fully evaluated.

In Japan, real-word data of NIVO+IPI with up to 1-year follow-up have been reported (17–19). We have also reported the 1-year analysis results of this retrospective observational study enrolling patients with mRCC treated with NIVO+IPI at nine Japanese institutions (J-cardinal study) (20): the effectiveness and safety of NIVO+IPI at 1 year after the last enrollment in the real-world setting were comparable with those in CheckMate 214. However, long-term follow-up data including data of patient populations excluded from phase 3 studies and effectiveness of second-line therapy are still required. In this paper, we report the results of 2-year analysis assessing the effectiveness of NIVO+IPI in patients with mRCC, including those with nccRCC, poor Eastern Cooperative Oncology Group (ECOG) PS, and without previous nephrectomy, and the effectiveness of second-line therapy.

## Patients and methods

### Study design

This study was a multicenter, retrospective, observational study conducted at nine hospitals in Japan. Data were retrospectively collected from patients’ medical records at baseline and at 3 months, 1 year and 2 years after the last enrollment. The study is registered with UMIN-CTR Clinical Trial under the title ‘Retrospective Japanese real-world study of mRCC treated with nivolumab plus ipilimumab (J-cardinal study)’ (ID: UMIN000035974).

### Patients

Adult patients, age ≥20 years, with IMDC intermediate or poor risk, previously untreated mRCC, who had initiated NIVO+IPI between 21 August 2018 and 31 January 2019, were allowed to enroll in this study.

### Ethics

This study was approved by the regulatory authorities and the ethics committee at each hospital and was conducted in compliance with Japanese Ethical Guidelines for Medical and Health Research Involving Human Subjects (21) and the Act on the Protection of Personal Information. All study procedures were conducted according to the principles of World Medical Association Declaration of Helsinki (22). All patients provided written informed consent and had the opportunity to withhold permission from researchers to use their medical records.

**Table 1:** Treatment patterns of nivolumab (NIVO) plus ipilimumab (IPI) combination therapy and second-line therapy

Category	n (%)
Treatment patterns of NIVO+IPI (*N* = 45)	
Ongoing NIVO+IPI combination therapy^a^	5 (11.1)
Discontinued NIVO+IPI combination therapy^a^	40 (88.9)
Reasons for NIVO+IPI discontinuation	
Progression	15 (33.3)
Adverse event	16 (35.6)
Discontinuation for efficacy	2 (4.4)
Withdrawal by subject	3 (6.7)
Death^b^	2 (4.4)
Others	2 (4.4)
Patient status after NIVO+IPI discontinuation	
Received second-line therapy	22 (48.9)
Treatment free	6 (13.3)
Untraceable	4 (8.9)
Death	8 (17.8)
Cancer death	3 (6.7)
Treatment related death^c^	3 (6.7)
Others	2 (4.4)
Treatment patterns of second-line therapy (*N* = 22)	
Reasons for NIVO+IPI discontinuation	
Progression	12 (54.5)
Adverse event	9 (40.9)
Others	1 (4.5)
Second-line therapy	
Axitinib	17 (77.3)
Sorafenib	1 (4.5)
Sunitinib	1 (4.5)
Others	3 (13.6)

^a^NIVO+IPI every 3 weeks for 4 doses followed by NIVO every 2 weeks.

^b^One was cancer death and the other death was related to treatment other than NIVO+IPI.

^c^Two were treatment (NIVO+IPI)-related deaths, the other death was related to treatment other than NIVO+IPI.

### Assessments

Objective response rate (ORR), overall survival (OS), PFS, disease control rate (DCR), treatment status of NIVO+IPI and effectiveness of second-line therapy after discontinuation of NIVO+IPI were documented in this study. This 2-year analysis did not collect safety data; therefore, the safety data have not been updated since the previous report (20). ORR was calculated as the proportion of patients who achieved complete response (CR) or partial response (PR) as best overall response (BOR) among patients with measurable disease at baseline, in accordance with Response Evaluation Criteria in Solid Tumors (RECIST) version 1.1 (23). Outcomes were reviewed by the researchers. DCR was defined as the percentage of patients with measurable disease in whom the BOR was either CR, PR or stable disease. PFS was defined as the period from the first dose to progression or death. OS was defined as the period from the first dose to death. PFS2 was defined as the period from the first dose of NIVO+IPI to death or progression of second-line therapy (8). Time to treatment failure (TTF) was defined as the period from the first dose to the final dose of NIVO+IPI. Treatment-free survival (TFS) was defined as the period from the final dose of NIVO+IPI to either death or start of second-line therapy.

Subgroup analyses based on baseline demographics and treatment history were performed to assess effectiveness.

### Statistical analysis

All assessments and baseline demographics were reported using descriptive statistics, and categorical variables were reported using number and percentage. The 95% confidence interval (CI) of ORR was calculated. OS and PFS rates with their respective 95% CIs at 2 years after the last enrollment were estimated using the Kaplan–Meier method. Subgroup analyses for OS were conducted by comparing the 24-month OS rates in Kaplan–Meier analysis. *P*-values were calculated by log-rank test, as applicable. *P* < 0.05 was regarded statistically significant. SAS version 9.1 or above (SAS Institute, Cary, NC, USA) was used for statistical calculations, including Kaplan–Meier method, 95% CI and hazard ratio (HR).

## Results

### Two-year follow-up

#### Patients

Patient demographics and baseline characteristics at the start of NIVO+IPI have been previously reported (Supplementary Table S1) (20).

#### Treatment patterns

**Figure 1:**
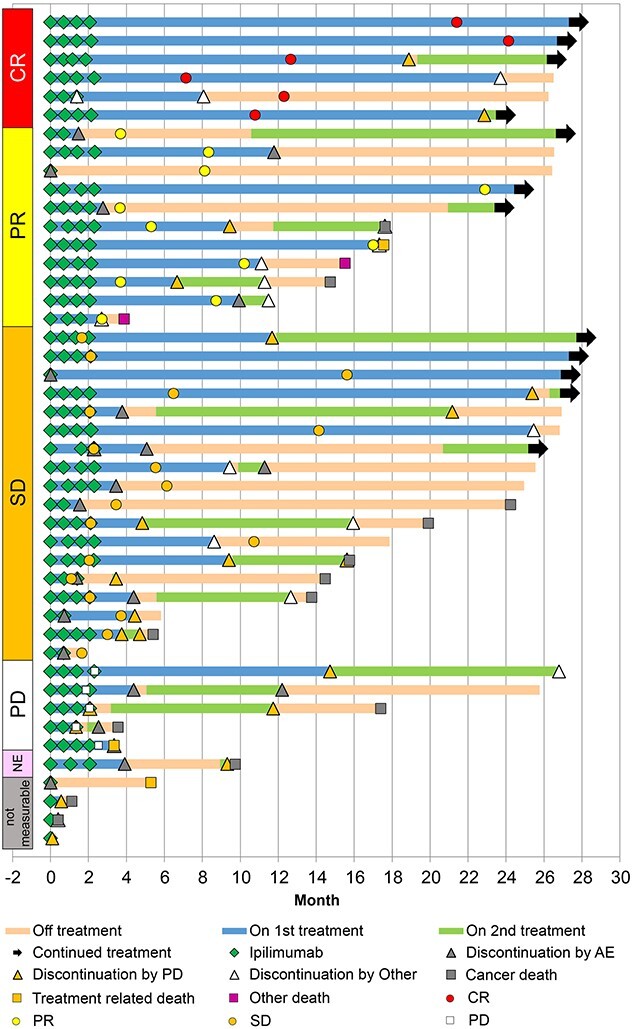
The time to failure of nivolumab plus ipilimumab combination therapy and second-line therapy, and treatment-free interval: The blue, green and orange bars show first-line treatment, second-line treatment and off treatment, respectively. *AE*, adverse event; *CR*, complete response; *NE*, not evaluable; *PD*, progressive disease; *PR*, partial response; *SD*, stable disease.

Table 1 shows the NIVO+IPI patterns in this real-world study. The median follow-up period was 24.0 (range, 0.3–28.3) months at the time of data cutoff. NIVO+IPI was ongoing for 5 patients (11.1%) and discontinued for 40 patients (88.9%). The major reason for NIVO+IPI discontinuation was disease progression in 15 patients (33.3%) and adverse events (AEs) in 16 patients (35.6%). The TTF and treatment-free interval are shown in Fig. 1. The median TTF was 4.5 months (95% CI, 2.8, 9.6). Six patients discontinued NIVO+IPI without second-line therapy. The median TFS was 1.9 months (95% CI, 1.0, 5.4).

Of the 22 patients treated with second-line therapy, 12 (54.5%) and 9 patients (40.9%) had discontinued NIVO+IPI due to disease progression and AEs, respectively, and 17 patients (77.3%) had received axitinib as second-line therapy.

#### Effectiveness

The antitumor activity of NIVO+IPI is summarized in Table 2, and 41 out of 45 patients had measurable disease at baseline. The ORR was 41.5% (95% CI, 26.3, 57.9), with 6 patients (14.6%) achieving CR. The DCR was 85.4% (95% CI, 70.8, 94.4). Compared with the 1-year analysis data (20), 2 patients with PR improved to CR status in the 2-year analysis. Of the 17 responders, 7 patients (41.2%) were durable responders at data cutoff (Fig. 1). The results of the subgroup analyses for BOR based on the baseline demographics and treatment history are shown in Supplementary Table S2.

**Table 2:** Antitumor activity of nivolumab plus ipilimumab combination therapy

	*N* = 41^a^
Objective response, *n* (%) [95% CI]	17 (41.5)
	[26.3, 57.9]
Disease control, *n* (%) [95% CI]	35 (85.4)
	[70.8, 94.4]
Best overall response, *n* (%) Complete response Partial response Stable disease Progressive disease Not evaluable	
	6 (14.6)
	11 (26.8)
	18 (43.9)
	5 (12.2)
	1 (2.4)

^a^Data were analyzed in 41 patients with measurable disease. The other 4 patients were excluded due to lack of measurable disease.

**Figure 2:**
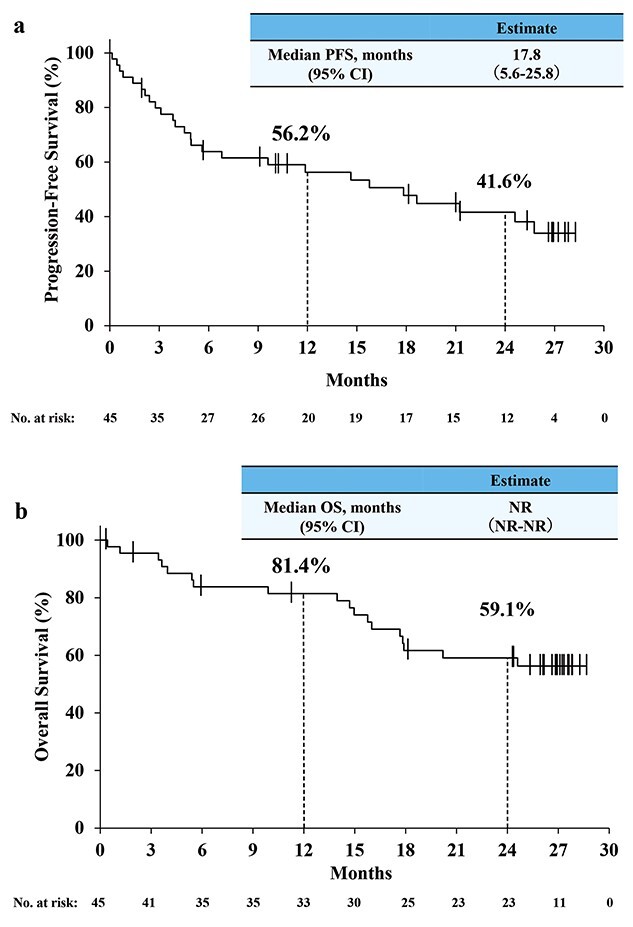
The Kaplan–Meier curves of (a) progression-free survival and (b) overall survival. *NR*, not reached; *OS*, overall survival; *PFS*, progression-free survival.

The Kaplan–Meier curves of PFS and OS are shown in Fig. 2a and b, respectively. The median PFS and the 24-month PFS rate were 17.8 months (95% CI, 5.6, 25.8) and 41.6% (95% CI, 26.0, 56.5), respectively. The median OS was not reached (NR; 95% CI, NR, NR) and the 24-month OS rate was 59.1% (95% CI, 42.6, 72.3). Subgroup analyses for OS based on patient demographics and treatment history were performed (Supplementary Fig. S1). The OS was analyzed with age (cutoff at 75 years), histology (clear cell RCC vs. nccRCC), previous nephrectomy, sarcomatoid differentiation, ECOG PS (0–1 vs. 2–3), IMDC risk group (intermediate vs. poor) and number of IPI doses (0–3 vs. 4). No significant differences except for IMDC intermediate and poor risk were observed.

### Second-line therapy

#### Patients

Patient demographics and baseline characteristics at the start of second-line therapy are shown in Table 3. Of the 22 patients treated with second-line therapy, 5 patients (22.7%) were aged ≥75; 15 patients (68.2%) had ECOG PS 0 or 1 and 11 patients (50.0%) were at IMDC intermediate risk. The numbers of patients with nccRCC, sarcomatoid differentiation or previous nephrectomy were 6 (27.3%), 2 (9.1%) and 8 (36.4%), respectively.

**Table 3:** Patient demographics and baseline characteristics at the start of second-line therapy

Category	*N* = 22
Gender, *n* (%)^a^	
Female	6 (27.3)
Male	16 (72.7)
Age, years	
Median (range)	68.0 (50–81)
<75, *n* (%)^a^	17 (77.3)
≥75, *n* (%)^a^	5 (22.7)
Weight, kg	
Mean ± SD	57.47 ± 10.96
ECOG PS, *n* (%)^a^	
0	10 (45.5)
1	5 (22.7)
2	3 (13.6)
3	1 (4.5)
Unknown	3 (13.6)
IMDC risk classification, *n* (%)^a^	
Intermediate	11 (50.0)
Poor	11 (50.0)
Number of risk factors for IMDC, *n* (%)^a^	
1	6 (27.3)
2	5 (22.7)
3	2 (9.1)
4	7 (31.8)
5	2 (9.1)
6	0 (0.0)
Histological type, *n* (%)^a^	
Clear cell carcinoma	16 (72.7)
Non-clear cell carcinoma	6 (27.3)
Papillary renal cell carcinoma	4 (18.2)
Unclassified	2 (9.1)
Sarcomatoid, *n* (%)^a^	
No	20 (90.9)
Yes	2 (9.1)
Previous nephrectomy, *n* (%)^a^	8 (36.4)
Number of metastasis sites, *n* (%)^a^	
0	1 (4.5)
1	13 (59.1)
2	7 (31.8)
≥ 3	1 (4.5)
Sites of metastasis, *n* (%)^a^	
Lung	15 (68.2)
Liver	0 (0.0)
Bone	6 (27.3)
Brain	0 (0.0)
Lymph node	5 (22.7)
Other	4 (18.2)

*ECOG PS*, Eastern Cooperative Oncology Group Performance Status; *IMDC*, International Metastatic Renal Cell Carcinoma Database Consortium.

^a^Percentage of patients was calculated with 22 as 100%, for patient demographics at the start of second-line therapy.

#### Effectiveness

**Figure 3:**
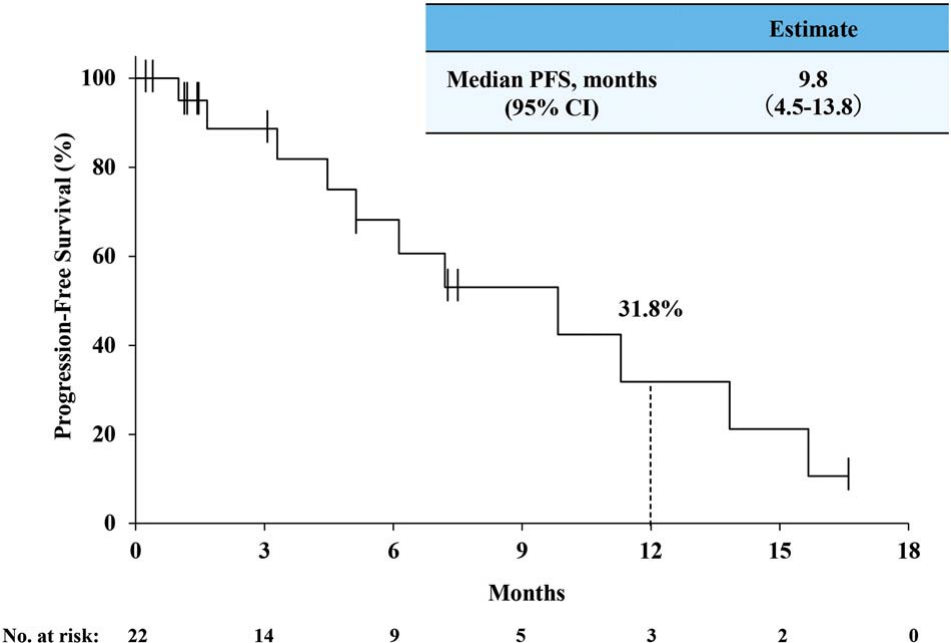
The Kaplan–Meier curve of PFS in second-line therapy.

**Figure 4:**
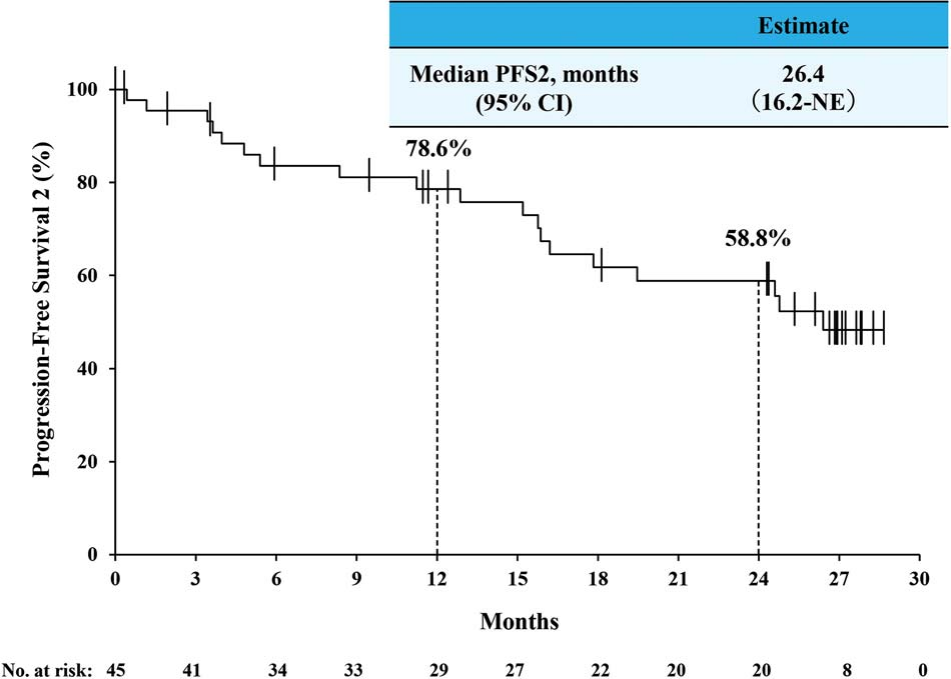
The Kaplan–Meier curve of PFS 2^*^. ^*^PFS 2 was defined as the period from the first dose of nivolumab plus ipilimumab combination therapy to progression or death during second-line therapy. *NE*, not estimable; *PFS2*, progression-free survival 2.

The antitumor activity of second-line therapy was evaluable among 15 out of 22 patients at data cutoff (Table 4). The ORR was 20.0% (95% CI, 4.3, 48.1), and the DCR was 80.0% (95% CI, 51.9, 95.7). Figure 3 shows the PFS Kaplan–Meier curve for second-line therapy. The median PFS of second-line therapy was 9.8 months (95% CI, 4.5, 13.8), and the 12-month PFS rate was 31.8% (95% CI, 8.7, 58.5). The median PFS2 was 26.4 months (95% CI, 16.2, not estimable), and the 12-month PFS2 rate was 78.6% (95% CI, 62.8, 88.3) (Fig. 4).

**Table 4:** Antitumor activity of second-line therapy

Category	*N* = 15^a^
Objective response, *n* (%) [95% CI]	3 (20.0)
	[4.3, 48.1]
Disease control, *n* (%) [95% CI]	12 (80.0)
	[51.9, 95.7]
Best overall response, *n* (%) Complete response Partial response Stable disease Progressive disease	
	0 (0.0)
	3 (20.0)
	9 (60.0)
	3 (20.0)

^a^Data were analyzed in 15 evaluable patients.

The effectiveness of axitinib as second-line therapy was as follows. The ORR, DCR, median PFS and median PFS2 were 25.0% (95% CI, 5.5, 57.2), 75.0% (95% CI, 42.8, 94.5), 9.8 months (95% CI, 4.5, 15.7) and 19.5 months (95% CI, 12.9, 26.4), respectively.

## Discussion

In this 2-year analysis, we have reported the effectiveness of NIVO+IPI in the actual clinical practice in Japan, which confirms the effectiveness of NIVO+IPI reported in the 1-year analysis (20). To our knowledge, this study is the first report to show the 2-year real-world data of NIVO+IPI in Japanese patients with mRCC. Several studies regarding NIVO+IPI in Japanese mRCC patients have been published (17–19); however, no study has reported the median follow-up of 2 years or more. In addition, we also found that the effectiveness of second-line therapy was comparable with that in the previous reports (12,13,16,24).

In this study, 2 patients with PR at the 1-year analysis improved to CR at the 2-year analysis. The CR rate (14.6%, Table 2) was numerically higher than that seen in CheckMate 214 (9%) (1) and was comparable to the real-world data of Tachibana et al. (13.0%) (18). All 6 patients with CR had ECOG PS 0, and the IMDC risk was poor in 1 patient and intermediate in the remaining 5 patients. The ORR (41.5%, Table 2) was comparable to that of CheckMate 214, and other Japanese real-world data (1,17–19,25). The median PFS (17.8 months, Fig. 2a) was numerically longer than that in CheckMate 214 (11.6 months) (1). The favorable CR rate and PFS, in fact, were a surprising finding, as the proportion of patients with IMDC poor risk (51.1%) was numerically higher in this study than in CheckMate 214 (21%) (1). Our study included a lower proportion of patients with 2 or more metastasis sites (37.8%) than that of CheckMate 214 (79%) (1). The proportion of patients with liver or lymph node metastasis in our study (8.9 or 24.4%) was lower than that of CheckMate 214 (21 or 45%) (1). Patients with multiple metastases or with liver metastasis tended to show worse response to NIVO+IPI than patients with single metastasis or without liver metastasis in some Japanese real-world data (17,26). These differences in metastasis may have influenced the favorable CR rate and PFS, despite the higher proportion of patients with IMDC poor risk. Kido et al. (27) also reported a better PFS (median PFS, 17 months) compared with that of CheckMate 214, with higher proportion of patients with IMDC poor risk (48%), and the Japanese real-world data show similar or better response to NIVO+IPI than that reported in the clinical trials.

The median OS (NR, Fig. 2b) and the 24-month OS rate (59.1%, Fig. 2b) of NIVO+IPI in this study were comparable to those in CheckMate 214 (NR, 66%) (28). In CheckMate 214, patients with liver or lymph node metastasis showed worse prognosis than patients without liver or lymph node metastasis (28). Consistently, the 24-month OS rates of patients with or without liver metastasis in our study were 33.3 or 61.0%, and those with or without lymph node metastasis were 40.0 or 65.2%, respectively. In addition, the proportion of patients treated with second-line therapy at 2 years in this study was 48.9% (Table 1), whereas that in CheckMate 214 was 39% at a median follow-up of 25.2 months (1). Compared with the actual clinical practice in the USA, where 37.9% of patients received second-line therapy including IO and tyrosine kinase inhibitor (TKI) therapies (29), the proportion of patients treated with second-line therapy after NIVO+IPI in this study was numerically higher. Furthermore, the most common second-line therapy in this study was axitinib (77.3%, Table 1), whereas the most common second-line therapy in CheckMate 214 was sunitinib (20%) (1). Not only the metastasis but also the second-line therapy may have contributed to the comparable 2-year OS compared with that of CheckMate 214.

CheckMate 214 did not include patients with nccRCC or poor PS (1). This study included 10 patients (22.2%, Supplementary Table S1) each in these categories, and the effectiveness of NIVO+IPI was confirmed regardless of histology or PS (Supplementary Fig. S1b and e). In CheckMate 920, which is a P3b/4 study evaluating NIVO+IPI in patients with backgrounds that were excluded in CheckMate 214, the median OS in patients with nccRCC was 21.2 months and the median OS in patients with Karnofsky performance-status score 50–60% was 15.6 months, which were both comparable to those of this study (nccRCC, 20.2 months; ECOG PS 2–3, 15.0 months) (30,31). Although TKI monotherapy has been reported to have a poor prognosis in patients with nccRCC and poor PS (32–36), this study suggested that the prognosis may not differ substantially from the overall population even in patients with nccRCC and poor PS, which were exclusion criteria in CheckMate 214. While Tachibana et al. and Bando et al. reported the real-world data of NIVO+IPI in patients with nccRCC (18,37), its effectiveness remains controversial. Further evaluation of IO combination therapies in patients with nccRCC is needed in larger studies.

This study included 18 patients with previous nephrectomy (40.0%, Supplementary Table S1) and demonstrated that the prognosis of NIVO+IPI was comparable, irrespective of previous nephrectomy (median OS, with previous nephrectomy: NR vs. without previous nephrectomy: 24.6 months; 24-month OS, 65.5 vs. 54.5%, respectively) (Supplementary Fig. S1d). After CARMENA and SURTIME studies, the number of patients treated with systemic therapy after cytoreductive nephrectomy has decreased (38,39). Compared with the period of CheckMate 214 (80% with previous nephrectomy) enrollment, the proportion of patients with previous nephrectomy has been decreasing (1,4,5), and this trend was also observed in this study. In CheckMate 214, the median OS of patients without previous nephrectomy was 26.1 months for NIVO+IPI versus 14.3 months for sunitinib (HR 0.63; 95% CI 0.40–1.0) at a minimum follow-up of 4 years (40), and both NIVO+IPI and sunitinib tended to show shorter OS in patients without previous nephrectomy than in the overall population (41). The prognosis of patients without previous nephrectomy was not poor in this study, which was inconsistent with that of CheckMate 214. These results suggested that the significance of cytoreductive nephrectomy should be reconsidered for better prognosis of patients with mRCC in the IO era.

We also examined the effectiveness of second-line therapy after NIVO+IPI. Although there are some effectiveness data for sequential therapy after NIVO monotherapy in actual clinical practice (10,11,15,16), effectiveness data of second-line therapy after NIVO+IPI remain scarce (12,13,16,24). The effectiveness of TT as second-line therapy after the first-line that includes TT was previously reported (ORR, 0–19%; median PFS, 4.7–9.3 months) (14,42–48), and so was effectiveness of TT following NIVO+IPI (ORR, 28.6–45%; median PFS, 8–16.3 months) (12,13,16,24). The ORR and PFS of TT as second-line therapy after the first-line that includes TT were lower than those of TT after NIVO+IPI. Second-line TT after NIVO+IPI may be effective due to the change in the mode of action between treatment lines, and patients treated with NIVO+IPI were TT naïve. In this study, the ORR (20%, Table 4) and PFS (median PFS, 9.8 months; Fig. 3) of TT after NIVO+IPI were similar or better than those of TT as second-line therapy after the first-line that included TT. The ORR and PFS of our data were lower than the previously reported TT after NIVO+IPI by Tomita et al. and Auvray et al. (12,13). Their results were based on patients from clinical trials, whereas our study does not include any patients from clinical trials. In our study, 11 patients (50.0%, Table 3) had IMDC poor risk and none had favorable risk at the start of second-line therapy, while in these previous reports, the proportion of poor risk was 12.5 and 21.2%, and the proportion of those at favorable risk was 6.3 and 15.1%, respectively (12,13). Higher proportion of patients with more risk factors may have affected the effectiveness of second-line therapy (35,49). Because this study showed similar effectiveness between axitinib and other second-line treatment, the topic that which TT is appropriate after NIVO+IPI needs further investigation. The effectiveness of second-line TT from this study should be interpreted with caution, because these data were obtained only from patients treated with TTs after NIVO+IPI discontinuation at data cutoff. Patients for whom NIVO+IPI was ongoing and patients without treatment after NIVO+IPI discontinuation were not analyzed for effectiveness of second-line TT.

For devising the treatment strategy of RCC from first-line therapy, it would be important to consider not only the effectiveness of the first-line therapy but also the outcome of sequential therapy, such as PFS2. However, PFS2 in IO combination therapies is scarcely reported. Tomita et al. (50) reported that patients who received TT after NIVO+IPI had a median PFS2 of 32 months. Although PFS2 of IO combination therapies was also reported in the JAVELIN Renal 101 (avelumab+axitinib) and CheckMate 9ER (NIVO+cabozantinib), the follow-up was short (minimum and median of 13 and 18.1 months, respectively), and the median PFS2 was NR (4,51). Consistently, the median PFS2 was 26.4 months in this study (Fig. 4), suggesting that a sequential TT after NIVO+IPI may have a clinical benefit.

The limitations in this study include the retrospective observational study design, small sample size, limited follow-up, lack of review by a central reviewer and limited number of institutions. Therefore, further studies are necessary to confirm the results of this study.

In conclusion, the effectiveness of NIVO+IPI in the real-world setting in Japan was comparable to that of reported in CheckMate 214 at 2 years after the last enrollment. The current analysis also demonstrated the effectiveness of second-line therapy after NIVO+IPI.

## Supplementary Material

J-cardinal_2nd_Paper_Supplementary_Tables_hyac124

J-cardinal_2nd_Paper_Figure_S1_hyac124
