# Supplementary material for: Real-world effectiveness of nivolumab plus ipilimumab and second-line therapy in Japanese untreated patients with metastatic renal cell carcinoma: 2-year analysis from a multicenter retrospective clinical study (J-cardinal study)
Source: Jpn J Clin Oncol. 2022 Aug 3;52(11):1345–52. doi: 10.1093/jjco/hyac124 (PMC9631464; doi:10.1093/jjco/hyac124)
Supplement: J-cardinal_2nd_Paper_Supplementary_Tables_hyac124 [file j-cardinal_2nd_paper_supplementary_tables_hyac124.doc]

# Supplementary Tables

**Table S1.** Patient demographics and baseline characteristics at the start of nivolumab plus ipilimumab combination therapy

| Category | N = 45 |
| --- | --- |
| Gender, n (%)^a^ |  |
| Female | 9 (20.0) |
| Male | 36 (80.0) |
| Age, years |  |
| Median (range) | 70.0 (50-85) |
| <75, n (%)^a^ | 32 (71.1) |
| ≥75, n (%)^a^ | 13 (28.9) |
| Weight, kg |  |
| Mean ± SD | 60.57 ± 14.55 |
| ECOG PS, n (%)^a^ |  |
| 0 | 22 (48.9) |
| 1 | 13 (28.9) |
| 2 | 8 (17.8) |
| 3 | 2 (4.4) |
| IMDC risk classification, n (%)^a^ |  |
| Intermediate | 22 (48.9) |
| Poor | 23 (51.1) |
| Number of risk factors for IMDC, n (%)^a^ |  |
| 1 | 6 (13.3) |
| 2 | 16 (35.6) |
| 3 | 12 (26.7) |
| 4 | 2 (4.4) |
| 5 | 6 (13.3) |
| 6 | 3 (6.7) |
| Histological type, n (%)^a^ |  |
| Clear cell carcinoma | 34 (75.6) |
| Non-clear cell carcinoma | 10 (22.2) |
| Papillary renal carcinoma | 5 (11.1) |
| Unclassified | 3 (6.7) |
| Other | 2 (4.4) |
| Unknown | 1 (2.2) |
| Sarcomatoid, n (%)^a^ |  |
| No | 40 (88.9) |
| Yes | 5 (11.1) |
| Previous nephrectomy, n (%)^a^ | 18 (40.0) |
| Number of metastasis sites, n (%)^a^ |  |
| 0 | 1 (2.2) |
| 1 | 27 (60.0) |
| 2 | 11 (24.4) |
| ≥3 | 6 (13.3) |
| Sites of metastasis, n (%)^a^ |  |
| Lung | 24 (53.3) |
| Liver | 4 (8.9) |
| Bone | 12 (26.7) |
| Brain | 0 (0.0) |
| Lymph node | 11 (24.4) |
| Other | 16 (35.6) |

^a^ The percentage was calculated with 45 as 100%.

*ECOG PS* Eastern Cooperative Oncology Group Performance Status, *IMDC* International Metastatic Renal Cell Carcinoma Database Consortium

**Table S2.** Antitumor activity by patient demographics and treatment history

| Variables | Category | N | n (%)^a^ | | |
| --- | --- | --- | --- | --- | --- |
|  |  |  | Complete Response | Objective response | Disease control |
| Overall |  | 41 | 6 (14.6) | 17 (41.5) | 35 (85.4) |
| Age | <75 years | 29 | 4 (13.8) | 10 (34.5) | 26 (89.7) |
|  | ≥75 years | 12 | 2 (16.7) | 7 (58.3) | 9 (75.0) |
| ECOG PS | 0 | 22 | 6 (27.3) | 11 (50.0) | 20 (90.9) |
|  | 1 | 13 | 0 (0.0) | 4 (30.8) | 9 (69.2) |
|  | 2-3 | 6 | 0 (0.0) | 2 (33.3) | 6 (100.0) |
| Histological type | ccRCC | 31 | 5 (16.1) | 13 (41.9) | 27 (87.1) |
|  | nccRCC | 9 | 1 (11.1) | 3 (33.3) | 7 (77.8) |
| Sarcomatoid | Yes | 4 | 0 (0.0) | 3 (75.0) | 3 (75.0) |
|  | No | 37 | 6 (16.2) | 14 (37.8) | 32 (86.5) |
| Previous nephrectomy | Yes | 18 | 5 (27.8) | 10 (55.6) | 14 (77.8) |
|  | No | 23 | 1 (4.3) | 7 (30.4) | 21 (91.3) |
| Number of IMDC risk factors | 1 | 6 | 2 (33.3) | 5 (83.3) | 6 (100.0) |
|  | 2 | 16 | 3 (18.8) | 8 (50.0) | 15 (93.8) |
|  | 3 | 11 | 1 (9.1) | 1 (9.1) | 9 (81.8) |
|  | 4-6 | 8 | 0 (0.0) | 3 (37.5) | 5 (62.5) |
| KPS | <80 | 6 | 0 (0.0) | 2 (33.3) | 6 (100.0) |
|  | ≥80 | 35 | 6 (17.1) | 15 (42.9) | 29 (82.9) |
| Hemoglobin | <LLN | 31 | 3 (9.7) | 10 (32.3) | 25 (80.6) |
|  | ≥LLN | 10 | 3 (30.0) | 7 (70.0) | 10 (100.0) |
| Corrected calcium | <10 mg/dL | 26 | 4 (15.4) | 11 (42.3) | 24 (92.3) |
|  | ≥10 mg/dL | 15 | 2 (13.3) | 6 (40.0) | 11 (73.3) |
| Duration from diagnosis to start of treatment | <1 year | 38 | 6 (15.8) | 15 (39.5) | 32 (84.2) |
|  | ≥1 year | 3 | 0 (0) | 2 (66.7) | 3 (100.0) |
| Neutrophil count | >ULN | 13 | 0 (0.0) | 3 (23.1) | 9 (69.2) |
|  | ≤ULN | 28 | 6 (21.4) | 14 (50.0) | 26 (92.9) |
| Platelet count | >ULN | 7 | 0 (0.0) | 2 (28.6) | 4 (57.1) |
|  | ≤ULN | 34 | 6 (17.6) | 15 (44.1) | 31 (91.2) |
| Number of IPI doses | 0-3 | 12 | 1 (8.3) | 4 (33.3) | 10 (83.3) |
|  | 4 | 29 | 5 (17.2) | 13 (44.8) | 25 (86.2) |

^a^ The percentage was calculated with each number of patients in each category as 100%.

*ccRCC* clear cell renal cell carcinoma, *ECOG PS* Eastern Cooperative Oncology Group Performance Status, *IMDC* International Metastatic Renal Cell Carcinoma Database Consortium, *IPI* ipilimumab, *nccRCC* non-clear cell renal cell carcinoma, *KPS* Karnofsky performance-status score*,* *LLN* lower limit of normal, *ULN* upper limit of normal
